# A Randomized Comparison of Hemoglobin Content-Based Versus Standard (Unit-Based) Red Blood Cell Transfusion Policy

**DOI:** 10.4274/tjh.2015.0365

**Published:** 2017-08-02

**Authors:** Erden Atilla, Selami Koçak Toprak, Sinem Civriz Bozdağ, Pervin Topçuoğlu, Önder Arslan

**Affiliations:** 1 Ankara University Faculty of Medicine, Department of Hematology, Ankara, Turkey

**Keywords:** Blood components, Blood processing, Donors, Transfusion strategy

## Abstract

**Objective:**

The hemoglobin (Hb) content of packed red blood cells (pRBCs) differs in standard volume units. The pRBC transfusions are based on the number of units routinely. We aimed to use pRBCs according to total Hb content and compare the rates of achieving the target Hb concentration levels with the current transfusion practice.

**Materials and Methods:**

Eighty-nine patients (55 males and 34 females) with median age of 46 years (range: 19-75) were enrolled, and of 178 transfusion episodes, 92 were randomized to the Hb content based-study group and 86 to the unit-based control group. Fifty-one patients were evaluated by 1 and rest of the patients by ≥2 episodes (median: 3; range: 1-7). Suitable pRBCs were detected by the Hemosoft Blood Banking Management & Information System. In the Hb content-based study group, to reduce the number of units, the required Hb was calculated by recipients’ height, weight, and Hb levels. When no appropriate units could be found within the inventory, the actual ordered number of units was sent to clinics, as was done for the control group.

**Results:**

In the study group totally, 38 units of pRBCs were transfused with a reduction of 19.8% (38/192) from the original order. The success of finding the matched Hb content was statistically increased with low weight and height and high pRBC storage. The Hb content of transfused pRBC units was significantly higher in the study group than the control group. The ratio of achieving the target Hb level was statistically similar in the control and study group (p=0.125), the successful and unsuccessful group (p=0.325), and the control and unsuccessful group (p=0.438). The relation between the shelf-life of the pRBC units and the rate of achieving the target Hb level was found to be similar between groups (p=0.782).

**Conclusion:**

The number of pRBC transfusions can be minimized since we clearly demonstrated that the efficacy of Hb content-based transfusion is similar to that of unit-based transfusion.

## Abstract (tr)

**Amaç:**

Eritrosit süspansiyonu (ES) ünitelerinin hacimleri standart kriterlerle belirlenmişse de hemoglobin (Hb) içerikleri farklıdır. Halen klinikteki standart transfüzyon uygulamaları, ES’lerin ünite sayısına dayanmaktadır. Bu çalışma ile Hb içeriğine dayalı transfüzyon ile standart ES transfüzyonunun etkinliğinin karşılaştırılması amaçlanmıştır.

**Gereç ve Yöntemler:**

Çalışmaya dahil edilen 89 hastanın (55 erkek; 34 kadın) ortanca yaşı 46’dır (aralık, 19-75). Çalışmada 92’si Hb içeriğine dayalı transfüzyon (çalışma grubu), 86’sı standart transfüzyon (kontrol grubu) olarak randomize edilen toplam 178 transfüzyon epizodu; 51 hasta için 1, 38 hasta için ≥2 epizotta değerlendirilmiştir (ortanca 3; aralık, 1-7). Uygun olan ES’ler, kan bankamızda kullanılan Hemosoft İşletim Sistemi kullanılarak belirlenmiştir. Çalışma kolunda, gerekli ünite sayısını ES-Hb konsantrasyonuna göre belirlemek amacıyla: alıcının boy, güncel kilo, güncel ve hedef Hb verileri ile alıcı için gerekli Hb miktarı hesaplanmıştır. Stokta doğru Hb konsantrasyonunda ES bulunamayanlarda aynen kontrol kolunda olduğu gibi, klinisyenin istediği sayıda ES ünitesi gönderilmiştir.

**Bulgular:**

Çalışma kolunda toplam 192 ünite ES transfüzyonu istemine karşılık Hb konsantrasyonuna göre uygun 38 ünite ES transfüzyonu gerçekleşmiş ve ES ünite miktarında %19,8 (38/192) oranında azalma gösterilmiştir. Uygun Hb içeriğinde ES ünitesi bulma ihtimalinin; düşük kilolu, kısa boylu hastalarda daha yüksek ve kan bankası ES stok sayısı ile doğru orantılı olduğu bulunmuştur. Transfüzyon yapılan ES ünitelerindeki Hb konsantrasyonları, çalışma grubunda kontrol grubuna göre daha yüksek olarak tespit edilmiştir. Hedeflenen Hb değerine ulaşma oranları; kontrol ve çalışma (p=0,125), uygun ünite bulunan ve bulunamayan (p=0,325), kontrol ve uygun ünite bulunamayan (p=0,438) gruplarda istatistiksel olarak benzer bulunmuştur. ES ünitelerinin raf ömürleri ile hedef Hb düzeylerine ulaşma arasındaki ilişki gruplar arasında benzer olarak saptanmıştır (p=0,782).

**Sonuç:**

Çalışmamız sonucunda açıkça görülmüştür ki Hb içeriğine dayalı transfüzyon ile standart ES transfüzyonlarının etkinlikleri benzerdir. Hb içeriğine dayalı transfüzyonlar ile kullanılan ES ünite sayısı azaltılabilir.

## INTRODUCTION

Currently, packed red blood cell (pRBC) concentrates are the major blood components transfused in routine medical practice. Despite numerous reliable scientific and physiological data, indications for pRBC transfusion are controversial. The decision to transfuse RBCs is based on the patient’s pulmonary, cardiovascular, and cerebrovascular statuses and expected duration of anemia [1]. Each unit of whole blood (WB) or pRBCs contains enough hemoglobin (Hb) to raise the Hb concentration of an average-sized adult by approximately 1 g/dL. Unfortunately, the total Hb content of the units does not represent homogeneity due to different Hb concentration levels of donors, Hb losses during component preparation processes (buffy coat isolation, leukofiltration), and storage. Although it has generally been accepted for many years that pRBC transfusions should be based on the Hb content of the product, instead of units, for practical reasons this has been implemented in few studies [2,3].

By using a computer program, Hemosoft, in a randomized study, we aimed to use pRBCs according to their total Hb content in order to decrease the number of units and to compare the rates of achieving the target Hb concentration levels with the current transfusion practice.

## MATERIALS AND METHODS

The present study was carried out as a prospective, randomized, open-label clinical study at the Hematology Clinic and Stem Cell Transplantation Unit and the Blood Banks of Ankara University between October 2010 and February 2011. The study protocol was approved by the Ankara University Faculty of Medicine Ethics Committee and written and signed informed consent was provided by all participants. The Hemosoft Blood Banking Management & Information System (version 2.0) used as part of this study is a newly developed in-house software system. It fulfills all American Association of Blood Banks and British Committee for Standardization in Haematology guidelines for blood-bank computing and information technologies [4].

A total of 89 consecutive patients were included in the study. A total of 364 pRBCs were ordered in 178 transfusion episodes. The median age and male-to-female ratio were 46 years (minimum: 19, maximum: 75) and 55:34, respectively. Patients’ diagnoses were acute leukemia in 44 cases (27 acute myeloblastic leukemia, 13 acute lymphoblastic leukemia, 3 acute myeloblastic leukemia secondary to myelodysplastic syndrome, and 1 acute lymphoblastic transformation of chronic myeloid leukemia), lymphoproliferative disease in 17 (8 non-Hodgkin’s lymphoma, 2 Hodgkin’s lymphoma, 6 chronic lymphocytic leukemia, and 1 hairy cell leukemia), plasma cell disorders in 11 (10 multiple myeloma and 1 Waldenström’s macroglobulinemia), bone marrow failure in 15 (12 myelodysplastic syndrome, 2 aplastic anemia, and 1 paroxysmal nocturnal hemoglobinuria), sickle cell anemia in 1, and solid tumor in 1. The transfusion policy was based on symptoms of anemia rather than low Hb levels. Patients with acute blood loss who required emergent blood component transfusions, those with autoimmune hemolytic anemia, and those younger than 18 years old were excluded from the study.

Initial assessment of donor Hb concentration was obtained by finger-stick puncture as part of the routine procedure at the donor sessions. A calibrated HemoCue hemoglobinometer (Hemo Control, EKF Diagnostics, Poland) was used for Hb determination. Hb lower cut-offs for women and men were respectively 12.5 and 13.5 g/dL. The upper cut-off limit for the Hb level was 18 g/dL. The WB collections were drawn into 450-mL triple bags with a standardized volume of 63 mL of citrate-phosphate-dextrose solution (Kansuk, Turkey). A saline-adenine-glucose-mannitol solution (100 mL; Kansuk, Turkey) was added to the RBC bag after the extraction of 200-250 mL of residual plasma by centrifugation from WB. The volumes of WB collected during phlebotomy and the pretransfusion Hb values of the donors were all recorded with Hemosoft as part of routine procedure. All units were leukoreduced by leukocyte filters (Pall, UK) in the laboratory before transfusion. Total Hb content of each pRBC unit in the inventory was calculated as “donor Hb level x 450 mL” automatically in Hemosoft.

Clinicians were asked to provide the height, actual body weight (ABW), and actual and target Hb level of each patient during pRBC orders. In the Hb content-based study group, based on the formula using total blood volume (TBV), Hemosoft directly calculated the Hb quantity required to achieve the target Hb and scanned the Hb contents of RBC units in the inventory: total Hb required to achieve targeted Hb (g) = (targeted Hb - actual Hb) g/dL x TBV. Using this approach, the goal was to find the best pRBCs from the inventory to decrease the number of units. When no appropriate units could be found within the inventory, the actual ordered number of units was sent to the clinics, as was done for the control group. Posttransfusion Hb level was checked 2 h after transfusion in both groups by using peripheral venous blood samples (Coulter STKS, USA).

### Statistical Analysis

Numerical variables were given as medians and distributions, which were compared with the nonparametric Mann-Whitney U test and the Kruskal-Wallis method. Nominal variables were compared by cross-table method using the chi-square test. P<0.05 was assumed to be statistically significant. SPSS 15.0 for Windows (SPSS Inc., Chicago, IL, USA) was used.

## RESULTS

A total of 178 transfusion episodes occurred in 89 patients, which were randomized into a control group (unit-based, n=86) and a study group (Hb content-based, n=92). The recipients within both groups had similar age, sex, body mass index, height, Hb level before transfusion, and targeted Hb level (Table 1). Fifty-one patients were evaluated for only 1 transfusion episode whereas 38 patients had 2 or more episodes (median of 3, ranging from 1 to 7). The patients’ ABWs and heights were also similar between the cohorts with 1 and ≥2 episodes (p=0.413 and p=0.956).

**Figure 1:**
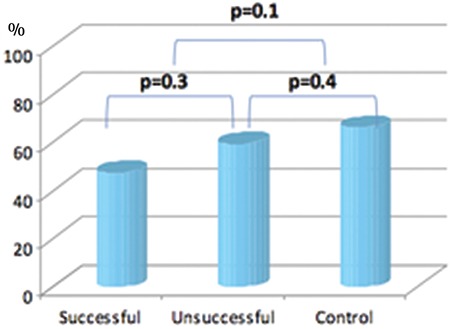
Comparison of rates of achieving targeted hemoglobin level.

The clinicians requested a total of 364 units of pRBCs within 178 transfusion episodes (n=170, 2 units per episode; n=8, 3 units per episode). Eighty-four of the 92 episodes in the study group were a 2-unit order and 8 were a 3-unit order. The rate of finding Hb content-matched pRBCs was 41.3% (38/92) and the rate of saving pRBC units was 19.8% (38/192) in the study group. In the case of no Hb content-matched units found in the study group, two pRBC units were given as ordered in the 54 episodes, respectively. All patients received two pRBC units in 86 transfusion episodes as requested by the clinician in the control group. The probability of finding Hb content-matched pRBC units was significantly higher in females and patients with lower body weight and shorter heights in the study group (p<0.0001) (Table 2). The rates of achieving targeted Hb levels were not different between the study and the control groups, nor between patients who successfully received Hb content-matched pRBCs and those not in the study group (Figure 1).

The median Hb level of the donors was 15.6 g/dL (minimum: 12.6, maximum 17.9 g/dL) and median Hb content of the products was 70.2 g (minimum: 56.7, maximum: 80.6 g). The Hb content of transfused pRBC units was significantly higher in the study group than the control group (p<0.0001) (Table 3). There was a positive correlation between the rate of achieving targeted Hb level and Hb content of each pRBC unit in both the study and control groups (p=0.001). This correlation was significantly marked in patients who successfully received Hb content-matched pRBCs in the study group (p=0.042).

The shelf-lives of pRBCs were similar in both the study and control groups (p=0.281) (Table 3). When we compared the shelf-life of pRBCs in the successfully Hb content-matched group and those not in the study group, we found no statistical difference (median of 6.5 days vs. 4 days, p=0.963). There was no correlation between the shelf-life of the pRBCs and the rate of achieving the targeted Hb level after the transfusion in the patients who successfully received Hb content-matched pRBCs (p=0.782).

The inventory distribution of pRBC units according to blood groups is given in Table 4. We found that the pRBC inventory size was larger in patients who successfully received Hb content-matched pRBCs than those who were not in the study group (77 units vs. 41 units, p=0.029) (Table 4). It was estimated that there should be a median of 93 units of pRBCs (33-180 units) in the blood bank inventory in order to transfuse Hb content-matched pRBCs with a success rate of 50%.

## DISCUSSION

The Hb content of pRBC units not being standardized can lead to the clinician’s irrelevance in terms of the number of units ordered. The factors responsible for this variation are the donor’s Hb concentration, loss of Hb during the preparation process, and shelf-life before transfusion [3,5]. To have a final standardized unit, different approaches have been used, such as apheresis devices for multicomponent collection. Depending on the donor’s Hb level and TBV, it is possible to collect either one or two units without exceeding 13% of any donor’s TBV, provided that the collected volume of blood in each unit is less than the current standard, which would allow reasonable use of the donor population. Two-unit blood collections may reduce donor exposure in transfusion. Applying a standard at 45 g of Hb per unit was found to permit the collection of maximum Hb and plasma in an evaluated population of Scandinavian donors [6]. An apheresis device was modified to facilitate the combined collection of a unit (250 mL) of RBCs and a high-volume unit (475 mL) of plasma [7]. The apheresis procedure was acceptable and well tolerated by donors, but the Hb content of the units differed with respect to Hb concentration of the donors. In our study, the Hb amount of the donor (minimum: 12.6, maximum: 17.9 g/dL) and the Hb content of pRBCs (minimum: 56.7, maximum: 80.6 g) also varied by 50%. The Hb content of transfused pRBCs was demonstrated to be significantly different in the study and control groups (p<0.0001) (Table 3). Holme et al. [8] stated that pRBC units collected by apheresis demonstrated low variability in volume of RBC mass collected and showed similar RBC properties as compared to manually collected ones after processing and 42 days of storage. Because the disposable kits for apheresis are currently more expensive than multiple-bag systems used for manual blood separation, Gilcher et al. [9] proposed an alternative approach for standardized units with a collection device by which a controlled volume of blood could be mixed in a specified proportion of anticoagulant. According to the donor’s predonation Hb concentration, this device would allow the collection of a volume of blood that contains the targeted RBC Hb mass. Finally, better-standardized RBC content, not depending on generation technique, helps to improve the accurate dosage used necessary for the recipient.

In the present study, the efficiency of transfusion was evaluated by the rates of achieving targeted Hb levels. The rates of achieving targeted Hb were not statistically different in the control and study groups (p=0.125) (Figure 1). Rates of achieving targeted Hb were similar in patients who successfully received Hb content-matched pRBCs and who did not (47.4% vs. 59.3%, p=0.325) (Figure 1). Rates of achieving targeted Hb levels were similar in the control group and the patients who did not receive Hb content-matched pRBCs in the study group (p=0.438) (Figure 1). Thus, we showed that transfusion policy based on number of units used in routine applications has similar efficiency to that of transfusion based on the product’s Hb content.

In the pilot study of Arslan et al. [2] in 2004 including 51 patients, it was demonstrated that transfusion policy based on Hb content reached target Hb levels with fewer numbers of transfusion units. Different from our current study, all patients were included with only one transfusion episode, with their rate of finding pRBC suspensions containing appropriate Hb content being 62.7% (32/51), and the rate of savings in transfused units was calculated as 30% (72 U/104 U) [2]. In our study, these rates were 41.3% (38/92) and 19.8% (38 U/192 U), respectively (Table 2). As a result, similar to the previous study of our team, it was shown that transfusion policy based on Hb content can lead to the saving of pRBC units.

In the study group, the subgroup that successfully received Hb content-matched pRBCs had a female predominance, lower ABW, and shorter stature (Table 2). These findings lead to our conclusion that for recipients with smaller body surface area, the chance of finding products with appropriate Hb content is higher. Our results also confirm the findings of Reikvam et al. [10]. The aim of their study was to evaluate whether the Hb increment in the patient can be predicted from the Hb dose transfused and their success rate was closely linked to the patient’s weight. In addition, both studies determined that the Hb content of transfused products was closely related to the reaching of target Hb levels.

In the present study, we determined that the major limitation of transfusion based on Hb content was finding pRBCs with appropriate Hb content. Because of this obstacle, limited numbers of transfusion episodes could be evaluated in both our studies and other publications. It is a fact that the increase in the rate of products with appropriate Hb content would increase the success of applications. Thus, we determined that the total number of pRBCs in the inventory, the height of the recipient, and the recipient’s current body weight are important parameters. It is known that inventory size is an important factor for finding the products with appropriate Hb content [3]. In the study of Arslan et al. [2], the relation between the inventory size at the time of request and the finding of the appropriate unit was not significant. However, in the present study, we found that pRBC inventory size was larger for the subgroup of patients who successfully received Hb content-matched pRBCs than those who did not in the study group (77 units vs. 41 units, p=0.025) (Table 4). Thus, we calculated that in order to achieve 50% success, the median pRBC units available in the inventory should be 93 (minimum: 33, maximum: 180).

Shelf-life is known as an important factor for the viability of the product. During ex vivo storage, red cells undergo changes affecting function and viability [11]. In the Red-Cell Storage Duration Study trial, researchers showed in 1098 patients that RBCs stored for 10 days or less were not superior to those with 21 days of duration in terms of Multiple Organ Dysfunction Score [12]. In the present study, shelf-life of the study and control groups was compared with similar results (p=0.281) (Table 3). In addition, shelf-life had no effect on targeted Hb after transfusion (p=0.782).

## CONCLUSION

We clearly demonstrated in the present study that the number of pRBC transfusions could be minimized by the rational use of the Hb content of the units. The subgroup that successfully received Hb content-matched pRBCs had female predominance, lower ABW, and shorter stature. Upon evaluation of the efficacy of transfusions based on Hb content of pRBC units, the success of this method related to reaching target Hb levels after transfusion was similar to the standard method based on clinicians’ orders. We indicated that the blood center’s inventory size was important for finding pRBC suspensions with sufficient Hb contents. Although the total number of patients who will benefit from this approach seems to be limited, it allows us to use units with high Hb contents rationally by using a computer system.

## Figures and Tables

**Table 1:**
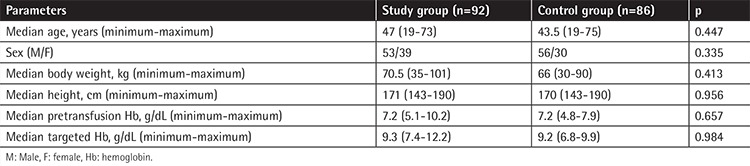
Characteristics of the patients in study and control groups.

**Table 2:**
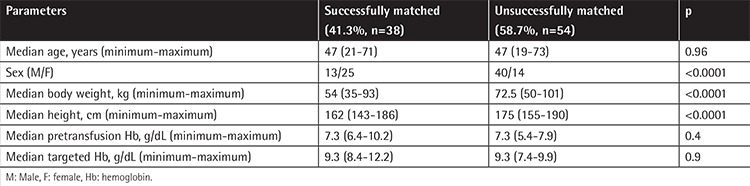
Comparison of successful versus unsuccessful matches in the study group.

**Table 3:**
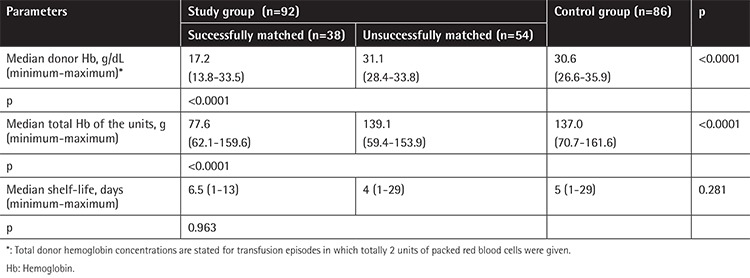
Comparison of shelf-life and hemoglobin contents of units within study and control groups.

**Table 4:**
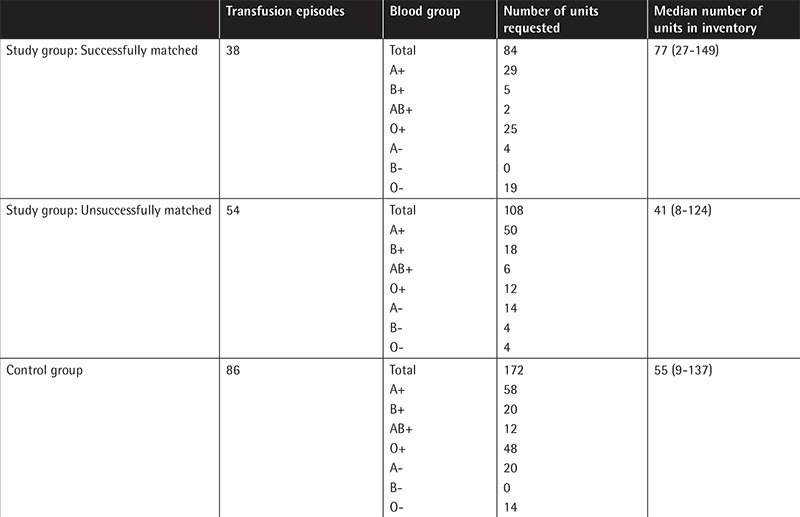
The inventory distribution of packed red blood cell units according to blood groups.
